# Neuroprotection by Radical Avoidance: Search for Suitable Agents

**DOI:** 10.3390/molecules14125054

**Published:** 2009-12-07

**Authors:** Rüdiger Hardeland

**Affiliations:** Johann Friedrich Blumenbach Institute of Zoology and Anthropology, University of Göttingen, Berliner str. 28, D-37073 Göttingen, Germany; E-Mail: rhardel@gwdg.de

**Keywords:** electron leakage, free radicals, melatonin, mitochondrial metabolism modifiers, nitrones

## Abstract

Neurodegeneration is frequently associated with damage by free radicals. However, increases in reactive oxygen and nitrogen species, which may ultimately lead to neuronal cell death, do not necessarily reflect its primary cause, but can be a consequence of otherwise induced cellular dysfunction. Detrimental processes which promote free radical formation are initiated, e.g., by disturbances in calcium homeostasis, mitochondrial malfunction, and an age-related decline in the circadian oscillator system. Free radicals generated at high rates under pathophysiological conditions are insufficiently detoxified by scavengers. Interventions at the primary causes of dysfunction, which avoid secondary rises in radical formation, may be more efficient. The aim of such approaches should be to prevent calcium overload, to reduce mitochondrial electron dissipation, to support electron transport capacity, and to avoid circadian perturbations. L-Theanine and several amphiphilic nitrones are capable of counteracting excitotoxicity and/or mitochondrial radical formation. Resveratrol seems to promote mitochondrial biogenesis. Mitochondrial effects of leptin include attenuation of electron leakage. Melatonin combines all the requirements mentioned, additionally regulates anti- and pro-oxidant enzymes and is, with few exceptions, very well tolerated. In this review, the perspectives, problems and limits of drugs are compared which may be suitable for reducing the formation of free radicals.

## Introduction

Damage by free radicals as a cause of neurodegeneration has been discussed in countless publications. With the progressive increase of average lifetime in numerous countries, the probability of acquiring such an age-related disorder is steadily rising. The classic suggestion of counteracting the precocious loss of neurons as a result of damage by reactive oxygen and nitrogen species has been to combat their deleterious actions by radical scavengers. Despite some positive results in animal studies, the epidemiological and clinical outcome with conventional antioxidants such as ascorbate, tocopherols or carotinoids has remained relatively poor [1,2,3,4,5]. Although a sufficient supply with antioxidant nutrients is, of course, a prerequisite of healthy aging, a surplus of these compounds, given at elevated doses as food additives, does not seem to be a promising strategy, at least not as long as these substances are exclusively acting as radical scavengers. Moreover, pharmacological concentrations of some antioxidants can cause undesired effects, such as redox cycling or formation of toxic dimers or other secondary products.

For logical reasons, detoxification of reactive intermediates already formed is not the only way of attenuating damage by free radicals. Their formation should likewise be regarded as a possible target of intervention. Lower rates of free radical generation, especially in aging individuals, would also reduce damage to molecules and cell death. This is the more important as both the formation of free radicals and the vulnerability to their devastating actions are progressively increasing by age [6,7,8,9,10,11]. However, a rise in radical formation should not only be seen as a cause of further damage, but also as a possible consequence of an otherwise initiated malfunction, e.g., by neuronal or cardiac overexcitation, calcium overload, ER stress, protein misfolding or reduced expression of relevant proteins which may end up in enhanced mitochondrial electron leakage [9,12,13]. Consequently, attempts of reducing cellular radical generation should not only focus on proximate goals related to the sites of formation, but also try to identify and counteract the processes leading to the underlying primary dysfunction. Therefore, an approach of simply detoxifying radicals already formed cannot be sufficiently successful, so that radical avoidance in the broadest sense should come into focus [14].

Multiple sources of free radical formation are known [15]. Some of these seem to be of particular importance. Myeloperoxidase-expressing leukocytes such as macrophages and neutrophils are specialized for oxidant-based bactericidal actions and their contribution to oxidative stress can be dominant during inflammatory reactions, but this is usually locally restricted to the site of inflammation. Additionally, the strongly elevated levels of nitric oxide, as occurrring in the course of an inflammatory response, can considerably contribute to cell stress. Although, in this case, the oxidative and nitrosative stress may be locally dramatic, it should not be considered to be of major importance for processes of normal aging, as far as this is related to the slow, lingering changes underlying a more or less continually progressing deterioration. Mitochondria may have a more general relevance as a source of free radicals during aging. Electron leakage from the electron transport chain (ETC), which will be discussed below in detail, represents a steady source of reactive intermediates and mitochondrial malfunction has potentially severe consequences for cellular energy supply and survival.

The role of mitochondria should not be misinterpreted in terms of earlier concepts, in which the generation of free radicals was directly related to damage of mitochondrial DNA (mtDNA). First, the mitochondrial chromosome is not that much vulnerable as previously thought, but rather covered to a considerable extent with proteins such as the mitochondrial transcription factor A (TFAM), a high mobility group (HMG) related molecule, which fulfills functions in nucleoid structure, damage sensing and mitochondrial replication [16,17,18,19]. Other proteins, including antioxidant enzymes, are bound to the mtDNA as well [19]. Moreover, pharmacological experiments, which have supported interpretations concerning a role of mitochondrial mutations, are far from real life during normal aging [20]. Investigations in mtDNA mutator mice revealed that the production of free radicals was not demonstrably increased during aging, compared to controls, although mitochondrial mutations had accumulated [21]. Therefore, the original assumption of mitochondrial mutations as a source of enhanced radical generation is not supported.

However, this conclusion does not mean that vicious cycles in the function of mitochondria do not exist. They are only not related to the number of mtDNA mutations. They rather have a dynamics different from slow aging processes, but may become critical for the function of a mitochondrion and the survival of a cell. Rises in radical formation can certainly cause damage to the ETC and, thereby, enhance electron leakage and lead to an excess of oxidants further impairing electron flux. Upon dissipation into other compartments, the rest of the cell can be affected as well. Therefore, a vicious cycle should only be understood as a self-enhancing feedback loop between electron leakage and causes of further leakage from the damaged ETC. A revised concept of a mitochondrial vicious cycle remains meaningful in the light of age-related rises in radical formation, as observed [6,7,8,9,10,11]. Radical avoidance would imply the maximally possible prevention of the initiation of such vicious cycles, even if they will never be fully suppressed in real life. Although aging should not be seen solely under the aspect of oxidative and nitrosative damage, and although reduced mitochondrial efficacy is not exclusively a matter of electron dissipation, an efficient attenuation of radical formation will, at least, contribute to healthy aging. The challenge is the search for suitable agents that may reliably and efficiently attenuate radical formation without reducing life processes within the cell.

## The Multiple Levels of Enhanced Radical Formation and Radical Avoidance

The formation of free radicals cannot be entirely prevented, nor is this desirable, since these reactive intermediates have their place in the normal functioning of an organism. Instead, their attenuation to physiologically favorable rates should be the aim of interventions. Therefore, the various causes of enhanced radical generation have to be first considered and discussed with regard to the possibilities of avoiding an excess of oxidative and nitrosative damage.

The reasons of rises in free radical formation are more complex than usually believed. A composite phenomenon that is regularly observed during aging is the deterioration of circadian rhythmicity. This observation, which has been made in several mammals including the human being, comprises reductions in the amplitude and progressive phase advances of various rhythmic functions [22,23,24,25,26,27], which may be ultimately followed by a gradual decomposition of the rhythmic time structure [28,29,30]. In particular, a decline in the nocturnal secretion of the pineal hormone, melatonin, is frequently observed [31,32,33,34,35]. At first glance, the circadian oscillator system may not appear to be related to the formation of free radicals. However, this seems to be the case. This connection goes beyond the rather banal correlations between metabolic activity and corresponding rates of mitochondrial electron leakage. Analyses of circadian clock mutants revealed that, in organisms as distant as fruit flies and Syrian hamsters, changes in the internal circadian phasing relative to the external light/dark cycle caused enhanced oxidative damage [36,37,38]. This was especially observed in short-period mutants, and also strongly expressed in the arrhythmic *Drosophila* mutant *per*^0^. More recently, *per*^0^ flies were shown to be more susceptible to oxidative stress induced by H_2_O_2_ than wild-type animals [39]. These observations concerning malfunctioning oscillators may be seen in relation to impairments of health as they are frequently observed in shift workers, who are repeatedly subjected to circadian phase shifts. Malcoordination of internal rhythms, as occurring under conditions of insufficient, disrupted or dysphased coupling within the circadian multioscillator system should be seen as a cause of enhanced radical generation. The extent of damage may correlate with the tissue-specific rates of oxidant formation. Oxidative modifications were most pronounced in the Syrian hamster Harderian gland [37], an organ which is particularly vulnerable because of its extraordinarily high physiological generation of free radicals [40]. Possibilities of intervention at the level of the circadian oscillator system should be sought in the strengthening of coupling to external time cues, e.g., by intense light in the morning, and in favoring signaling by chronobiotics, such as melatonin. The intention should be to maintain a desirable internal coordination and high amplitudes of rhythmic functions.

Another aspect of enhanced radical generation is that of neuronal overexcitation, especially in connection with calcium overload. Glutamate toxicity can be largely explained on this basis, including the secondary rise in the formation of nitric oxide (NO) [41,42,43,44]. The vulnerability to calcium imbalance and excitotoxic insults via NMDA receptor activation can be greatly enhanced in a neurodegenerative disorder like Huntington’s disease. The mutated huntingtin, which misfolds because of an extended polyQ repeat, leads to both a change in iron metabolism and a calcium-dependent mitochondrial dysfunction characterized by impaired activities of complexes II and III, oxidative stress and decreases in the proton potential, which may end up in apoptosis [45,46,47,48,49,50,51,52]. Although the individual outbreak of Huntington’s disease may be difficult to prevent, and although not any detail of the disease is covered by this outline, the disorder sheds light on the role of calcium in radical generation and possibilities of attenuation by antiexcitatory agents.

Excitotoxicity is also a severe problem in brain inflammatory diseases. While an acute inflammation in the CNS will require primarily the combat against the infection, aspects of, first subclinical and eventually atypical, inflammation can be associated with neurodegenerative disorders, too. Alzheimer’s disease provides an example for such an atypical, lingering form of inflammation, in which some classical hallmarks such as neutrophil infiltration and edema are usually absent [53,54], whereas other characteristics including acute-phase proteins and cytokines are clearly demonstrable [53,54,55,56,57,58]. Moreover, NO, which plays roles in both neuronal excitation and inflammatory signaling, is enhanced and leads to damage by free radicals derived from its metabolite peroxynitrite, such as •NO_2_, hydroxyl (•OH) and carbonate radicals (CO_3_•^–^) [53,54,59]. Activation of microglia is demonstrable [60,61,62,63], and similar observations have been made in most other neurodegenerative disorders [62,63]. This has multiple consequences, since the activation of microglia does not only lead directly to the formation of reactive oxygen species and, via •NO, a free radical of moderate reactivity, to more dangerous peroxynitrite-derived radicals, but also to the formation of endogenous excitotoxins, in particular, quinolinic acid. Inflammatory signals, especially but not exclusively IFNγ, induce microglial indoleamine 2,3-dioxygenase [64,65,66,67,68,69,70]. This enzyme, which preferentially catabolizes tryptophan to *N*-formylkynurenine, a precursor of several other metabolites. One of these is quinolinic acid, a potent activator of NMDA receptors [71,72,73,74]. Enhanced formation of quinolinic acid is, in fact, evident in Alzheimer’s [66,68,69] and also Huntington’s disease [75,76].

As shown in the case of •NO, enzymatic formation of free radicals can be of particular relevance. In the brain, both neuronal and inducible NO synthase isoforms (nNOS and iNOS, respectively), can become critical with regard to cytotoxicity. In terms of damage by radical reactions, the secondary radicals formed from peroxynitrite are of higher importance than •NO itself. iNOS is mostly upregulated in microglia and astrocytes by immunological or stress-related signaling, whereas nNOS, especially in glutamatergic neurons, depends on excitation-dependent calcium entrance. Several enzymes are capable of forming reactive oxygen species (ROS). Among these, an important role is attributed to the isoforms of NAD(P)H oxidases (Nox), which contribute to superoxide formation in a quantitatively substantial manner [77,78,79,80,81,82]. Some of them are membrane-bound enzymes. They respond to various signals, but their metabolic regulation is not fully understood. Inductions of Nox isoforms have been repeatedly observed in various situations of stress, including oxidative stress, and also during aging [77,78,79,80,81]. Even social stress was shown to enhance Nox2 expression [82]. Upregulation of some Nox isoforms, such as Nox1, can stimulate microglial NO formation [79]. Collectively, these findings indicate that a positive feedback loop—in cybernetic terms—between primary ROS production because of other reasons and Nox stimulation leads to further increases in the formation of oxidants, nitrosating and nitrating intermediates. Therefore, radical avoidance should be possible by interfering with the primary causes of oxidative stress. Interestingly, one of the isoforms, Nox4, has recently been shown to be mitochondrially located, and was assumed to be identical with subunit IV of complex IV (cytochrome oxidase) [83].

Mitochondria as a major source of free radicals are particularly in the focus with regard to both rises in oxidant formation and the potential for radical avoidance. Originally, electron leakage from the ETC had been mainly related to changes between respiration states, such as state 3 or 4 respiration. Although this is certainly of relevance, our considerations should actually be more directed towards the appearance of bottlenecks in the ETC [84,85,86], along with the surprisingly and unexpectedly high dynamics of electron flux. The demonstration of superoxide flashes [87,88] clearly shows that the electron transport is not at all a steady, continual process which may be modulated, but in a smooth, gradually changing fashion. It rather reminds of a traffic control in which the lights are switching and some overflow into the side alleys takes place, so that the burst-like mitochondrial electron leakage may appear as a consequence of a stop and go phenomenon. Superoxide flashes have been observed at elevated rates under specific conditions of pathophysiological relevance, such as anoxia/reoxygenation [88], which is known to cause oxidative stress.

Electron leakage can occur at different sites of the ETC. Mostly, leakage from complexes I and III has been studied. In complex I, the iron-sulfur complex N2, which is located at the amphipathic ramp extruding to the matrix, has been identified as the site of electron leakage [89,90,91,92,93]. The alternative of direct electron transfer to oxygen vs. indirect transfer via mediation by semiquinones [89,90,94] has been a matter of controversy, but now tends to be in favor of the direct process [93]. Because of the localization of N2, superoxide anions are preferentially released from complex I at the matrix side. Electron leakage from complex III has been attributed to electron bifurcation from ubiquinol [95] and, more recently, to the Qo site, from where electrons are released to reduce oxygen especially when the intramonomer electron transfer between the two *b*_L_ hemes is interrupted [96]. Electron dissipation and, thus, superoxide anion formation occurs at complex III on both sides of the inner mitochondrial membrane [97]. Superoxide formation from complex IV has been rarely investigated directly, but the recently suggested identity of Nox4 with a subunit within this complex [83] would imply a significant contribution of this respirasome to radical formation. Whether this is really so, remains to be confirmed. In epithelial cells, a homocysteine-stimulated translocation of Nox4 into mitochondria was reported and related to levels of oxidant formation [98]. Such results would not easily be compatible with the structural role of the subunit and the assumed regulation may not be applicable to neurons. An additional aspect of electron leakage has arisen recently by relating superoxide flashes to the opening of the mitochondrial permeability transition pore (mtPTP) [88]. Previously, mtPTP opening was predominantly seen in connection with a fatal breakdown of the mitochondrial membrane potential and the initiation of the intrinsic apoptotic pathway, whereas this may now appear to be a regularly occurring phenomenon of rather regulatory nature, as long as the depolarization is sufficiently short. Nevertheless, these findings shed light on the dynamics of mitochondrial function, the burst-like formation of superoxide and, secondarily, on its oxidant and nitrating products.

Another aspect of mitochondrial dynamics that has recently emerged concerns the structure and intracellular distribution of these organelles in their relationship to oxidative stress and ETC dysfunction. In various neurodegenerative diseases, such as Alzheimer’s, Parkinson’s and Huntington’s diseases and amyotrophic lateral sclerosis, the balance between mitochondrial fusion and fission is severely disturbed [99,100,101]. In the course of disease progression, mitochondria are becoming shorter and mitochondrial density decreases preferentially in the periphery of the neurons, a change that is correlated with the loss of spines at the neurites. These findings are in accordance with the observation that, especially in Alzheimer’s disease, the expression of fusion-promoting proteins, such as DLP1 (=Drp1), OPA1, Mfn1 and Mfn2, is reduced, whereas the fission-promoting protein Fis1 is upregulated [99,100,101]. Correspondingly, overexpression of amyloid precursor protein (APP) in hippocampal neurons caused mitochondrial fragmentation and decreases in the number of peripheral mitochondria [99]. In M17 cells overexpressing APP, fusion was slowed, mitochondria disappeared from the remote areas, and a progressively declining number of these organelles was associated with a rise in ROS formation, reduced ATP generation and a lower mitochondrial membrane potential [99,102]. In cultured cortical neurons, knockdown of the fusion proteins DLP1 or Mfn2 led to mitochondrial fragmentation, as might be expected, but also to cell death [103]. All these findings underline the importance of mitochondrial structure and distribution for the functioning and survival of neurons. The relationship between oxidative stress and prevailing mitochondrial fission may be a mutual one. Prooxidant agents, including Aβ peptide, may initiate a disturbance in the balance between fusion and fission, but an otherwise induced prevalence of fission may cause oxidative damage and ATP deficiency as well.

The attenuation of free radical formation at the mitochondrial level, including the interconnections with Nox isoforms, should be regarded as a major aim of preventing excessive damage. This includes pathophysiological conditions as well as the normal processes of aging. However, the problem cannot be successfully tackled by simplicist concepts. On the one hand, respiration—and thus, to a certain extent, electron leakage—is related to metabolic rates, which have to be adapted to the momentary requirements. On the other hand, continued metabolic inactivity may be source of maladaptations, which can weaken the regulatory capacity and favor the occurrence of stress, already upon moderate physical or mental exercise. In fact, rises in oxidative stress are observed both after strenuous exercise and as a consequence of physical inactivity [104]. Therefore, an adaptive or, in other words, hormetic, application of moderate exercise has been suggested to minimize the occurrence of and susceptibility to oxidative stress [104,105,106,107]. Of course, the consequences of mild physical and mental training are complex, and involve various other aspects, such as effects on immune functions, circulation, and also neurogenesis.

Nevertheless, there is a general agreement about an age-related decay of mitochondrial functions, which is additionally aggravated in neurodegenerative disorders [7,9,11,20,54,84,85,86,106,108,109,110,86,106,108]. The initial reasons for these processes can be multiple ones and are not easily discernible in an individual case. A few aspects shall be summarized here. Rises in electron leakage result from bottlenecks in the ETC. Several causes of insufficient electron conductance through the ETC can be distinguished. Both •NO and its metabolite, peroxynitrite, can block the iron-containing components of the respirasomes [85,86,111,112,113]. •NO can directly act as an iron ligand or cause the formation of *S*-nitrosothiols, including the conversion to other nitrosating NO congeners or N_2_O_3_. Soluble *S*-nitrosothiols, such as *S*-nitrosocysteine or *S*-nitrosoglutathione, are known to transnitrosate protein thiols in ETC complexes [85,86,114,115]. Complex I is particularly vulnerable to S-nitrosation, which leads to enhanced superoxide formation at this site [115]. Peroxynitrite (ONOO^–^), which can be generated at substantial rates, because of the abundantly available O_2_•^-^, acts mainly via its secondary radicals, formed from either its protonated form (ONOOH → •NO_2_ + •OH) or its CO_2_ adduct (ONOOCO_2_^–^→ •NO_2_ + CO_3_•^–^) [85,86,116,117,118,119,120,121,122]. Both nitration and oxidation reactions can lead to respirasome dysfunction. This may affect the protein subunits of the respiratory complexes, but can also result from peroxidation of cardiolipin, which is required for the structural integrity of especially complexes III and IV [123,124,125,126]. Cardiolipin has additional effects on complex I and the prevention of electron leakage from this site [127]. It interacts with cytochrome c, thereby gaining a peroxidase activity that has further consequences for progressing cardiolipin peroxidation and cytochrome c release [128,129,130,131,132]. Imbalance of electron feeding between complexes I and II is another source of electron overflow, especially at complex I, and an excess of succinate can increase electron leakage by an order of magnitude [85,86,133]. Bottlenecks at complex III by internal disruption or at complex IV, as occurring under conditions of oxygen deficiency, will lead to electron dissipation at complex III [85,86]. Moreover, cytochrome C is subject to acetylation [134], a modification assumed to enhance electron dissipation, an effect reverted by SIRT5 [135]. Additionally, Nox4-related superoxide formation may depend on the functioning of complex IV and, thus, be related to oxygen availability. Therefore, optimal ETC function should also be a matter of oxygen availability and, therefore, blood circulation. The relationship between oxygen supply and mitochondrial function may be critical under some conditions, such as ischemia/reperfusion or atherosclerosis, if compromised mitochondria produce an excess of oxidants, while NO formation is upregulated, partially in attempts of improving blood supply, thereby leading to peroxynitrite and radicals deriving thereof.

Especially in aging tissues, further aspects of mitochondrial dysfunction may become relevant. Decreases in the expression of mitochondrial proteins, including the nuclear-encoded respirasome subunits, are assumed to cause declines in respiratory efficiency. In senescence-accelerated SAMP8 mice, eight protein subunits (NDUAA, NDUBA, NDUB7, NDUS1, NDUS3, NDUV1, ETFA, and UCRI) of respiratory complexes were shown to be downregulated [136]. Moreover, decreased complex I and IV activities were reported [137]. These studies were performed in the liver and results may be tissue-specific. In the brain, the changes have not yet been analyzed in such detail, but all investigations unanimously state that SAMP8 mice exhibit an advanced development of mitochondrial dysfunctions, characterized by a decreased respiratory control index, low efficiency in ATP synthesis, higher state 4 but lower state 3 respiration, and increased electron leakage [138,139,140,141]. Moreover, complex I and IV activities in brain mitochondria have been related to neurological performance [142].

Insufficiencies in cell respiration, as observed during aging, may also result from a decline in mitochondrial biogenesis. Therefore, a higher mitochondrial mass relative to the cell would facilitate an efficient use of respiratory substrates, thereby avoiding an elevated electron throughput per surface of the inner mitochondrial membrane and, thus, a lower frequency of electron overflow leading to superoxide formation. Consequently, the stimulation of mitochondrial biogenesis may be a means for reducing radical formation and this strategy has, in fact, come into focus of gerontological research [143,144,145,146,147,148].

## Nitrones

**Figure 1:**
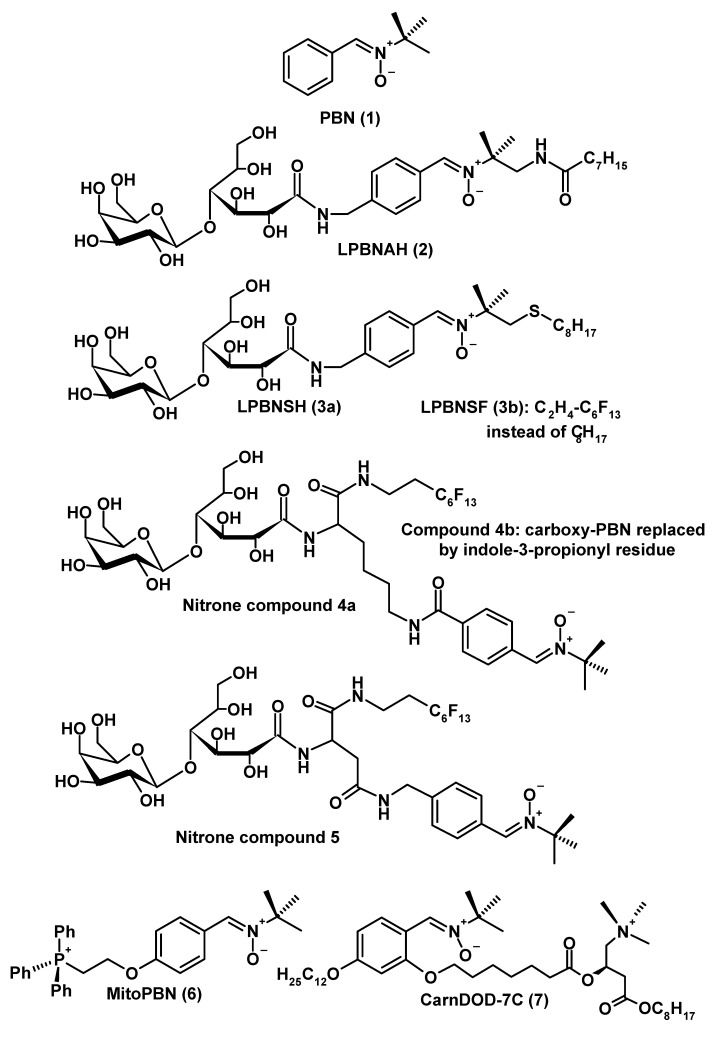
Several nitrones and a structurally related indolic compound, which have been tested for cell protection and attenuation of mitochondrial free radical formation. For details see current text.

Nitrones can exert antioxidant effects, but they differ from classic, electron/hydrogen-donating radical scavengers, such as ascorbate or tocopherols, by acting as spin traps [149,150,151]. The capability of trapping radicals goes beyond reactions with ROS and includes organic thiyl radicals, such as the glutathiyl radical [152]. The properties of the most frequently used nitrone, α-phenyl-*tert*-butyl-nitrone (PBN, **1**, Figure 1), has been extensively investigated and numerous neuroprotective effects have been ascribed to this compound [149,150,151,153,154,155,151,153]. In the course of many studies on the protection by PBN (**1**), it became apparent that the effects were not generally explained by a stoichiometric elimination of free radicals by the spin trap. Additional effects were identified, such as stimulation of Erk and Src protein kinases [156], blocking death pathways [157], inhibition of iNOS [150,158], downregulation of inflammatory mediators [150,158,159,160,161] and, most importantly, modulation of mitochondrial metabolism [150,155,162,163,164,165]. Despite the usefulness of PBN (**1**) in various animal models, several problems emerged with regard to clinical application. One of these difficulties is related to the limited stability of this compound, which can decompose to *N*-*tert*-butylhydroxylamine [166,167] and also liberate NO [168,169]. Moreover, PBN adducts can be additionally metabolized by P_450_ isoforms [170]. Although *N*-substituted hydroxylamines are usually believed to possess some toxicological potential [171], *N*-*tert*-butylhydroxylamine was reported to be protective against mitochondrial damage and to delay aspects of senescence more efficiently than the parent compound, PBN (**1**), so that the hydroxylamine derivative was assumed to represent the pharmacologically active agent in the mitochondria [166,167]. This conclusion contrasts to some extent with the observation that *N*-alkylated hydroxylamines inhibit glucose 6-phosphate dehydrogenase and glutathione reductase [171], two enzymes of relevance for the glutathione-based part of the mitochondrial protection system, but it may be, in the balance, correct. An additional aspect of protection by *N*-*tert*-butylhydroxylamine concerns its inhibitory effect on age-related iron accumulation [172], a finding that would require further substantiation in long-term *in vivo* studies. The consequences of NO release from decomposing PBN may be dose-dependent. In high concentrations as generated under conditions of inflammation, NO is certainly detrimental, as discussed above. However, NO was also reported to be protective at low concentrations. Even beyond its obvious value in ischemia, beneficial effects have been described, which are also related to mitochondrial function. Scavenging of free radicals other than superoxide anions, such as hydroxyl radicals, were reported [173,174], but this may be judged critically because of simultaneous formation of peroxynitrite by interaction with the more abundant superoxide. However, NO was also shown to act, in PC12 cells, as an antiapoptotic agent, via activation of guanylate cyclase and the PI3 kinase/Akt pathway [175]. cGMP-mediated protection by NO had been also observed in another study in PC12 cells [176] and in embryonic motor neurons [177]. Antioxidant actions of NO, associated with preservation of mitochondrial integrity, were reported in a study in astrocytes [178]. Whether or not modulation of antioxidant enzymes and other proteins of the mitochondrial protection system by NO, via PGC-1α (= peroxisome proliferator-activated receptor coactivator 1α) [179] is only an endothelium-specific mechanism, remains to be clarified. This reservation should be also made in another study conducted in the epithelium-derived cell line ECV304 [180]. In summary, the beneficial effects of NO appear rather conditional, are certainly restricted to low levels of this molecule, and this may equally apply to its liberation from PBN (**1**).

The relative instability of PBN (**1**) may be also the cause of either moderate toxicity and paucity in gerontoprotective efficacy that is sometimes observed with this compound [181,182]. Moreover, efforts were made to improve the bioavailability of nitrones especially to mitochondria. A major strategy was directed to the enhancement of amphilicity by attaching various suitable substituents [182,183,184,185,186,187,188,189]. A selection of these compounds (**2–7**) is presented in Figure 1. In particular, several mitochondria-specific approaches were recently undertaken by developing MitoPBN (**6**) and a carnitine-derived nitrone, CarnDOD-7C (**7**), that accumulates in these organelles by virtue of the carnitine-acylcarnitine translocase [189]. Several of the amphiphilic nitrones developed were shown to be superior to PBN (**1**), with regard to radical trapping, mitochondrial protection and life extension in model systems. One of these nitrones, LPBNAH {**2**; *N*-[4-(octa-O-acetyllactobionamido-methylene)benzylidene]-*N*-[1,1-dimethyl-2-(N-octanoyl)amido]-ethylamine *N*-oxide} may serve as an example, for which relatively many details are known [182,183,186]. The compound **2** was by many fold superior to PBN (**1**) in preventing cell death induced by H_2_O_2_, peroxynitrite or doxorubicin in mixed cortical cultures, it was considerably more effective in protecting SK-N-SH human neuroblastoma cells against lethal stress by H_2_O_2_, Aβ_1-42_ peptide, or the oxidotoxin 3-hydroxy-kynurenine, and, in submitochondrial particles from rat brain, it inhibited the inactivation of the iron-sulfur cluster N2 in complex I, as induced by H_2_O_2_, peroxynitrite or doxorubicin. Nanomolar concentrations of LPBNAH and a related nitrone, LPBNH15, were sufficient to decrease electron and proton leakage as well as hydrogen peroxide formation in isolated rat brain mitochondria [190].

In a gerontological model organism, the rotifer *Philodina acuticornis*, LPBNAH (**2**) was the by far most effective nitrone in protecting against toxicity by H_2_O_2_ or doxorubicin. A most impressive effect was observed in promoting longevity in otherwise untreated *Philodina*. Both mean and maximal lifespan were extended by 5 µM LPBNAH (**2**) by a factor of about 2.5, whereas PBN was only marginally effective [182]. A few other substituted nitrones exhibited, in some of these systems, a protective potential also exceeding that of PBN, such as *N*-lactobionyl-*N*^ε^-(*N*-*tert*-butyl-α-carboxy-phenylnitrone)-L-lysinyl-1*H*,1*H*,2*H*,2*H*-perfluorooctylamide (**4a**) and *N*-lactobionyl-β-(*N*-*tert*-butyl-4-amidomethylphenylnitrone)-L-aspartyl-1*H*,1*H*,2*H*,2*H*-perfluorooctylamide (**5**) [185]. Experience with the residues promoting amphilicity also prompted the development of similarly substituted indolic radical scavengers, such as *N*-lactobionyl-*N*^ε^-[3-(1H-indol-3-yl)propionyl)]-L-lysinyl-1*H*,1*H*,2*H*,2*H*-perfluorooctylamide (**4b**) [185]. All these compounds were more effective scavengers of hydroxyl radicals than PBN. Because of the presence of an indolyl moiety, compound **4b** was also capable of acting as an efficient reductant of ABTS cation radicals [ABTS = 2,2’-azino-*bis*-(3-ethyl-benzthiazoline-6-sulfonic acid)]. Of course, the demonstration of protection or life extension in cultured cells, isolated mitochondria and invertebrate model organisms can never replace long-term studies in mammals. Anyway, no prolongation of lifetime as observed in *Philodina* can be expected in a mouse or a human being. In fact, there is a lot of work ahead for testing the suitability of the most powerful nitrones, in terms of stability, toxicity, and possible side effects. For the moment, the studies mentioned should be rather understood as a proof of principle. To date, promising results in a mammalian system were obtained with an LPBNAH-related nitrone, LPBNSH (**3a**; *N*-{[4-(lacto-bionamido)methyl]benzylidene}-1,1-dimethyl-2-(octylsulfanyl)ethylamine *N*-oxide), which turned out to be highly effective in suppressing symptoms in a rat model of copper-induced hepatitis with jaundice [187]. In terms of the dose required, LPBNSH (**3a**) was by a factor of 500 – 1,000 more potent than PBN (**1**). It will be of great interest to see whether this newly developed compound will also be suitable for neuroprotective purposes.

## Leptin

The possibly beneficial effects by moderate levels of NO, as already discussed in the previous section, receives some support by other studies concerning leptin. Leptin is usually regarded as a regulator of appetite, and, indirectly, a restriction of calorie intake may have its own value in supporting longevity. This relationship to calorie restriction is in accordance with many data, but has been usually associated with other mechanisms [143,147]. A contribution by leptin may be considered in the future. However, the actions of leptin exceed the first-discovered role in appetite regulation via control of orexin release. Signaling by leptin has been reported to be a major source of circulating NO [191], which is also cycling in a circadian fashion [38]. Moreover, leptin now appears to act as a more generalized modulator of metabolism, with orchestrating functions, and especially to modify mitochondrial activity [182]. This seems to include signaling by NO and its downstream factors. Leptin expression and secretion is not restricted to the classic sources, such as adipose tissue. This factor has been shown to be released by neurons, also in culture [193]. Moreover, leptin receptors are expressed in various brain regions, however, outside the hypothalamus in usually lower densities [194,195,196,197]. A novel promoter of the leptin receptor gene preferentially acting in neuronal cells has been recently identified [198]. The extrahypothalamic actions are poorly understood, but may not be irrelevant at all. Leptin-deficient rats and mice were impaired in their capability of spatial learning [199]. When leptin was administered in low concentrations, learning, memory performance, and *in vitro* long-term potentiation were facilitated [200]. In these stimulatory processes, NMDA receptors, Ca^2+^ and CaM kinase II were involved. However, higher leptin levels were reported to suppress long-term potentiation and sensitivity of NMDA receptors [200]. Whether or not this reflects the duality of either activating or inhibitory NO actions remains to be clarified.

Since work on leptin was, and continues to be, strongly focused on aspects of nutrition and obesity, the consideration of its possibly neuroprotective potential is still in its infancy. In primary neuronal cultures from embryonic rat brain, glucose/oxygen/serum-deprived cells used a model of ischemia were shown to upregulate leptin, along with several other factors such as neurotrophins [193]. Moreover, cell protection was shown to require not only these regulators, but also NO, in moderate concentrations. Effective neuroprotection was not only associated with the factors mentioned, but also with PPARγ and -α (=peroxisome proliferator-activated receptor-γ and -α) [193]. Signaling via PPARs may represent a cross-connection to other neuroprotective factors, as will be discussed in a following section. Leptin is known to act via AMP-activated protein kinase (AMPK), a regulator also implicated in aspects of longevity and mitochondrial biogenesis [201,202], and the downstream factors Jak-2 (=Janus kinase 2), STAT3 (=signal transducer and activator of transcription 3), as well as the feedback regulator SOCS3 (=suppressor of cytokine signaling 3) [196,197,203,204]. This signaling network contains a further cross-connection, as both AMPK and NO are activators of the PPARγ coactivator-1α (=PGC-1α) [202].

Direct protection by leptin, as observed in hippocampal neurons, was associated with the stabilization of the mitochondrial membrane potential, decrease in oxidative stress, upregulations of the mitochondrial MnSOD (manganese superoxide dismutase) and the antiapoptotic protein Bcl-xL [196]. Another, recently communicated aspect of neuroprotection relating leptin to mitochondrial functions concerns the mitochondrial uncoupling protein-2 (UCP2), the neuronal subform of UCPs [205]. The association of leptin receptors with UCPs had already been stated earlier, but had first been interpreted only in terms of thermogenesis and its control [206]. With regard to the wide distribution of UCPs in many tissues and organisms including plants [207,208], their function has to exceed thermogenesis. Mammalian UCPs can be controlled by leptin [205,209,210]. In SH-SY5Y neuronal cells, leptin induced UCP2 and was reported to protect from toxicity by MPP^+^ (1-methyl-4-phenylpyridinium), with regard to survival, maintenance of the mitochondrial membrane potential and ATP formation [205]. Surprisingly, no changes were found in ROS generation. Similar findings were reported by the same group for transfected SH-SY5Y cells overexpressing UCP4 [211]. The overexpressed transgene protected against MPP^+^ toxicity and, in this study, reduced oxidative stress. Moreover, respiration and ATP levels were not only maintained, but increased [211]. These findings contrast considerably with the biological function of UCPs normally assumed, which would consist in decreases of membrane potential and ATP synthesis, as known since long from brown adipose tissue. The issue is highly controversial and most interpretations are based on real uncoupling of proton flux leading to decreases in mitochondrial membrane potential and ATP, and, as usually assumed, reduced ROS formation, too [209,210]. The precise mechanism by which UCPs attenuate radical formation would certainly require further elucidation. Nevertheless, overexpression studies in animal models and data from knockouts indicated that UCP expression can prolong lifespan, as reviewed in refs. [209,210]. However, the question remains as to whether high UCP levels can cause or mimic calorie restriction in animal studies and, thereby, act via additional signaling pathways. This may be the case in investigations on longevity, but certainly not in acute pathophysiological experiments, as shown in mice, in which overexpression of UCP2 was clearly neuroprotective in an ischemia model [212].

While the debate on the mechanisms will go on, there is good reason to continue searching for protection and radical avoidance on the basis leptin signaling and modulation of mitochondrial proton flux. However, the situation is complicated under various aspects, especially because of the interconnections with calorie consumption/restriction, insulin levels and signaling, and leptin effects via NPY [213]. If practical applications in humans are intended, the use of leptin mimetics [214] may be also considered in the future.

## Thiazolidinediones

The consideration of thiazolidinediones may be unexpected, but several cross-connections exist to other mechanisms relevant to neuroprotection and mitochondrial function. The involvement of PGC-1α and PPARγ in protective mechanisms based on modulation of mitochondrial metabolism has already been mentioned in the previous section. These important regulators are implicated in other signaling pathways. PCG-1α, being a stimulator of mitochondrial biogenesis, is a substrate of the sirtuin subform SIRT1, the gene product of an important aging suppressor gene, which has been also associated with calorie restriction [143,147]. At PCG-1α and PPARγ, signaling by SIRT1 and its activators, such as resveratrol, by lipoic acid, p38 MAP kinase [215], NO, leptin and downstream factors like AMPK [202] converge.

**Figure 2:**
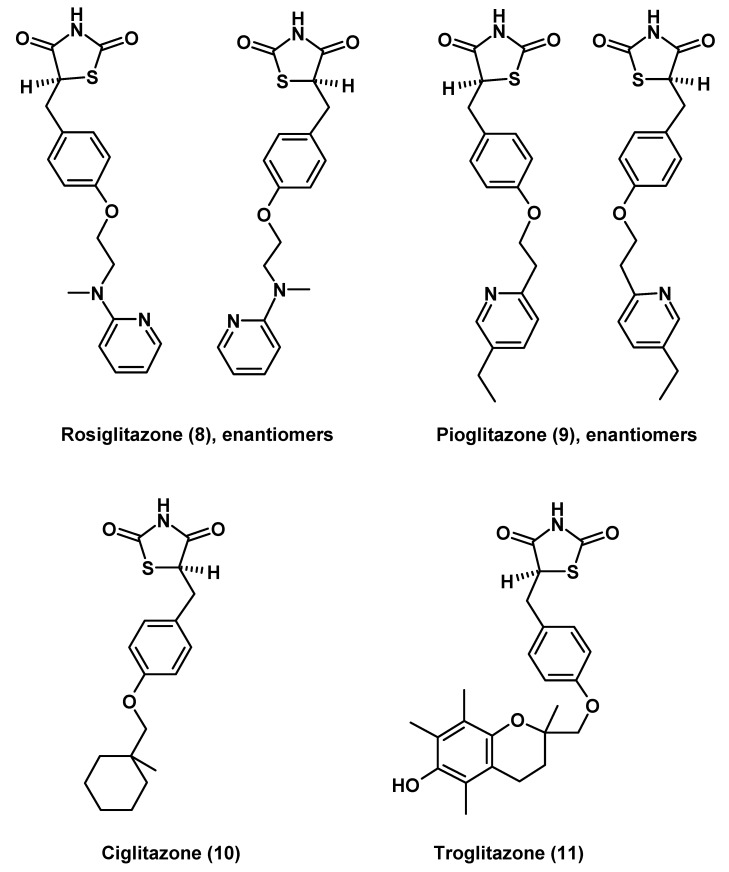
PPARγ activating thiazolidinediones with potentially neuroprotective and anti-neuroinflammatory properties.

Thiazolidinediones have been developed for the treatment of diabetes, and they have been shown to reduce insulin resistance [216,217]. Their use in neurological disorders has emerged more recently. Basis of these considerations is the primary action of this class of drugs, the activation of PPARγ, in the absence of similar affinity to other PPAR isoforms. Several compounds have been developed (**8**–**11**; Figure 2). Some of them, such as rosiglitazone (**8**) and pioglitazone (**9**), have entered the market, whereas others are to date of only used as investigational drugs, such as ciglitazone (**10**), rivoglitazone and MCC-555, or have been withdrawn, as in the case of troglitazone (**11**). Since PPARγ plays a role in numerous cells, the effects observed are countless, but the role in mitochondrial function may be crucial, especially in effects beyond diabetes. The suitability of thiazolidinediones in neurological diseases and neuroprotection should largely depend on their capability of crossing the blood brain barrier (BBB). The was considered to be poor by some investigators [218], but sufficient by others [219]. Especially under conditions enhanced BBB permeability, e.g., because of brain inflammatory diseases, the efficacy of thiazolidinediones should be higher. Therefore, thiazolidinediones were suggested for the treatment of brain inflammation including AIDS [219,220]. However, these studies were aimed to improve the function of leukocytes and endothelial cells, and are only indirectly related to neuroprotection. Nevertheless, attenuation of inflammation by rosiglitazone (**8**) was also observed after surgical brain injury [221], and pioglitazone (**9**) favored locomotor recovery after spinal cord injury [222]. Additional indirect, but not unimportant effects were described for rosiglitazone (**8**), which reversed cerebral hypertension by normalizing venous hydraulic conductivity and permeability [223].

More direct neuroprotective effects were reported for pioglitazone (**9**), which reduced infarct size after transient ischemia in rats [224], and rosiglitazone (**8**) in Alzheimer patients, a study that was, however, based on relatively few individuals [225,226,227]. Beneficial effects of rosiglitazone (**8**) on the preservation of memory were also reported in an Alzheimer mouse model [228]. A similar preservation of cognitive functions were obtained with telmisartan, a partial PPARγ activator structurally different from thiazolidinediones [229,230], otherwise known as an angiotensin II receptor antagonist. A protective role of PPARγ activation in Huntington’s disease was suggested in a study on cells expressing the mutant huntingtin [51]. In this case, rosiglitazone (**8**) prevented mitochondrial dysfunction and oxidative stress, when cells were challenged by elevated calcium. Beneficial mitochondrial effects on mitochondria were also described for ciglitazone (**10**) in another model using endothelial cells [98].

The mentioned studies should be mainly taken as an indication for the neuroprotective potential of PPARγ rather than the suitability of thiazolidinediones, especially with regard to long-term treatment. Apart from the question of limited bioavailability of thiazolidinediones in the central nervous system, this category of drugs is associated with several side effects, including body weight gain, congestive heart failure and ischemic cardiac events [231]. In the US, black box labels are warning about these side effects of the approved drugs rosiglitazone (**8**) and pioglitazone (**9**). Troglitazone (**11**) was withdrawn from the market because of its hepatotoxicity, which is, however, unrelated to PPARγ, but rather associated with the incorporation of a tocopherol-like component into the molecule (Figure 2) leading to a reactive metabolite [232,233]. Despite these limits, the consideration of neuroprotective and especially mitochondrial effects by thiazolidinediones should be of value for mechanistic reasons and may be recalled if other PPARγ activators having a different pharmacological profile will be developed in the future.

## Glutamatergic Modulators

**Figure 3:**
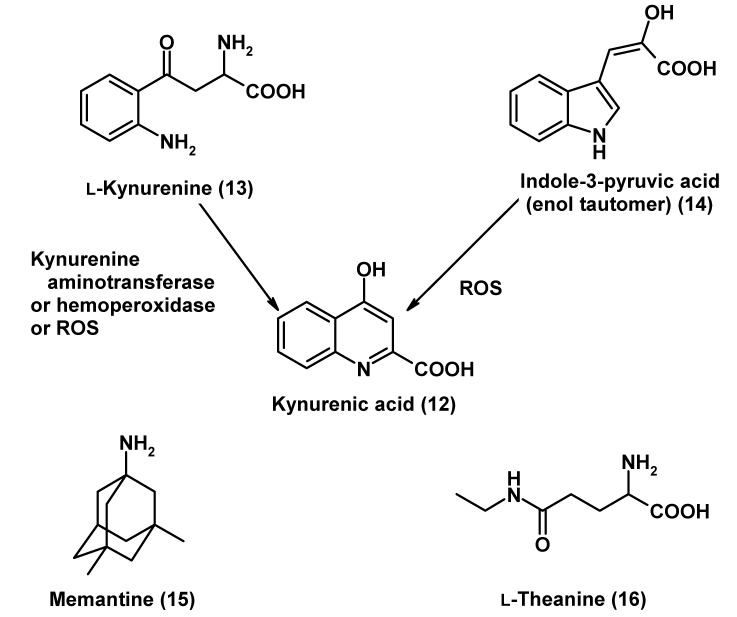
Glutamatergic modulators with antiexcitotoxic properties, and the kynurenic acid (**12**) precursors.

As outlined in the section on levels of radical avoidance, glutamate excitotoxicity is associated with calcium overload and NO overproduction. It represents a cause of mitochondrial dysfunction, oxidative stress and cell death. This can become locally critical under pathophysiological conditions in Huntington’s [75,76,234] and also Parkinson’s diseases. A physiologically formed NMDA modulator, kynurenic acid (**12**; Figure 3), has been especially studied in the striatum [235,236]. This tryptophan metabolite is formed and released by astrocytes [236] and acts by inhibiting the NMDA receptor by binding to the coagonist glycine_B_ site [236,237,238]. Additionally, kynurenic acid (**12**) decreases extracellular glutamate levels by blocking the presynaptic nicotinic receptors α7nAChR at glutamatergic nerve endings, an effect that is observed at nanomolar concentrations and has secondary consequences for dopamine levels [239,240]. The classic metabolic pathway of kynurenic acid (**12**) formation is that by transamination of L-kynurenine (**13**), a molecule at which tryptophan metabolism is branched and can also lead to the oxido- and excitotoxins L-3-hydroxykynurenine and quinolinic acid. These undesired effects are particularly important under brain inflammatory conditions, when the tryptophan-degrading enzyme indoleamine 2,3-dioxygenase is upregulated in the microglia under the influence of IFNγ, as discussed above. A changed balance between the excitotoxins and the antiexcitotoxic antagonist can be decisive in pathophysiological situations. Kynurenic acid (**12**) can be also formed in additional pathways, e.g., by oxidative deamination of L-kynurenine (**13**) under the influence of hemoperoxidases or by interactions with ROS [241]. Moreover, it can derive non-enzymatically via ROS from another tryptophan metabolite, the enol tautomer of indole-3-pyruvic acid (**14**), which is a very potent radical scavenger [241,242].

A problem with the practical use of kynurenic acid (**12**) for medicinal purposes is its poor penetration through the BBB. Therefore, other means have been sought to increase the intracerebral concentration of this metabolite. One approach consists in the use of kynurenine hydroxylase inhibitors, such as Ro61-8048 [236], thereby changing the flux of tryptophan catabolism in favor of kynurenine transamination. While this works well in an experimental preclinical design, toxicological considerations may speak against this method. However, another possibility exists by administering indole-3-pyruvic acid (**14**). In rodents, this precursor was shown to act as an anxiolytic and to shorten ethanol anesthesia, indicating a sufficient uptake into the brain [243,244,245]. In clinical studies (pilot trials and phase II) [246], indole-3-pyruvic acid (**14**) was very well tolerated, acted as an anxiolytic, promoted sleep quality, and did not show withdrawal effects. One should assume that these effects were largely due to actions mediated by its metabolite, kynurenic acid.

An entirely different compound that has also been used as a neuroprotective glutamatergic modulator is memantine (**15**; Figure 3), a substituted adamantane. Memantine (**15**) is a low-affinity voltage-dependent non-competitive antagonist of NMDA receptors, which reduces Ca^2+^ influx. It has been studied as an antiexcitotoxic agent [247] and tested and approved for the treatment of Alzheimer’s disease [248,249]. The compound is not an entirely selective NMDA antagonist, but acts additionally as a moderate inhibitor of serotonin 5-HT_3_ [250], nicotinic α7nAChR [251] and agonist at dopamine D_2_^High^ receptors [252]. Under experimental conditions, memantine (**15**) was capable of protecting against neurotoxic damage by methamphetamine or 3,4-methylendioxymethamphetamine (MDMA) and reduced the formation of ROS [251]. The double antagonism on NMDA and α7nACh receptors is functionally reminiscent of the effects by kynurenic acid (**12**). In terms of protection, the contribution of α7nAChR inhibition seems to have been the prevailing effect in the case of memantine (**15**) [251]. The general experience has been that memantine (**15**) is well tolerable, in both preclinical experiments and clinical trials [249]. However, its efficacy in the treatment of Alzheimer’s disease has remained relatively poor [249]. This should not be misinterpreted as a fundamental paucity as a protective agent, since the clinical success of other drugs is also rather limited, especially as the disease is usually diagnosed at a stage at which it cannot be halted or even easily delayed.

Another glutamatergic modulator that has been tested in experiments on excitotoxicity and with the purpose of attenuating radical formation is the glutamate analog L-theanine (=*N*-ethylglutamine; **16**; Figure 3). This natural compound present in the tea plant and some mushrooms has been approved in the US and in Japan as a food supplement and is usually regarded as being well-tolerated. It can sufficiently cross the BBB [253]. Despite its structural relationship to glutamate, its affinities to NMDA, AMPA and kainate receptors are in micromolar range and, thus, relatively low [254], but nothing else should have been expected for a compound present in a beverage used everyday by millions of people. Nevertheless, it exerts moderate sedating effects—which are outbalanced in the tea by caffeine—and several changes in neurotransmitter concentrations have been reported, such as rises in GABA and dopamine [254] and variable effects on serotonin [254,255]. Despite the poor effects on ionotropic glutamate receptors and although L-theanine (**16**) is not a radical scavenger, neuroprotective effects by this compound were described [254,256,257,258,259]. Interference with metabotropic glutamate receptors [254] or glutamate transporters [256] was speculated to be responsible for the effects observed. Nevertheless, L-theanine (**16**) was reported to decrease the size of cerebral infarcts in a mouse model [257]. In SH-SY5Y cells, high concentrations (0.5 mM) counteracted apoptosis, as induced by rotenone and dieldrin, and prevented declines in the formation of the neurotrophins BDNF and GDNF [258]. The antagonism to rotenone strongly indicates effects of L-theanine (**16**) at the mitochondrial level, although these should be of entirely indirect nature. Beyond its primary action as a cholinesterase inhibitor [260], which should be hardly relevant in cultured SH-SY5Y cells, dieldrin is excitotoxic and oxidotoxic [258]. In fact, neuroprotective, antiexcitotoxic and secondary, indirect mitochondrial effects have been also observed in other studies. This has been briefly mentioned under comparative aspects in a review on melatonin [261], but was unfortunately not yet published in detail by the investigators. In brief, L-theanine (**16**), studied in Fischer 344 rats, Swiss mice, and primary cortical neurons, prevented aging-related declines (24 months) in cognitive performance, in hippocampal neuronal density (CA1 and CA3), preserved hippocampal serotoninergic innervation, supported neurotrophic effects in culture, reduced striatal neurotoxicity by 3-hydroxykynurenine, and protected mitochondrial complex IV (B. Poeggeler, pers. commun.). Although the body of evidence for beneficial effects by L-theanine (**16**) is still rudimentary, and although the understanding of underlying mechanisms is insufficient, the compound seems worth further consideration.

## Resveratrol and Other Sirtuin Activators

Sirtuins are actually in the focus of gerontological research. These proteins, which act as NAD^+^-dependent *N*-deacetylases of lysinyl residues, are pleiotropic regulators of numerous cellular processes and, via activating PGC-1α by deacetylation and thereby targeting PPARγ, control a host of nuclear receptors and other signaling molecules [262,263,264]. Sirtuins are known to promote longevity in phylogenetically distant organisms, from yeast to insects and vertebrates. Moreover, at least one of them, SIRT1, has been related to caloric restriction [143,147,265]. In mammals, seven subforms have been described, SIRT1 to SIRT7, which are multiply involved in mitochondrial function. SIRT3, SIRT4 and SIRT5 are, or can conditionally be, mitochondrially localized [266]. The functional relationship of sirtuins to mitochondria does not depend on their presence within these organelles, but extends to additional regulatory effects and mitochondrial biogenesis. SIRT1, which is not mitochondrially localized, is also connected to free radical metabolism by modulating NO formation and the insulin/IGF-1 pathway, activating FoxO subforms and, thereby, stimulating antioxidant enzyme expression [147,267]. Both SIRT1 and AMPK simultaneously respond to elevated AMP and act concordantly in situations of stress, starvation or calorie restriction [268]. These actions result in elevated ATP formation, improved mitochondrial electron transport capacity and mitochondrial biogenesis [147,267]. This latter effect strongly depends on PGC-1α, which is an important stimulator of mitochondrial growth [202,215,269,270].

**Figure 4:**
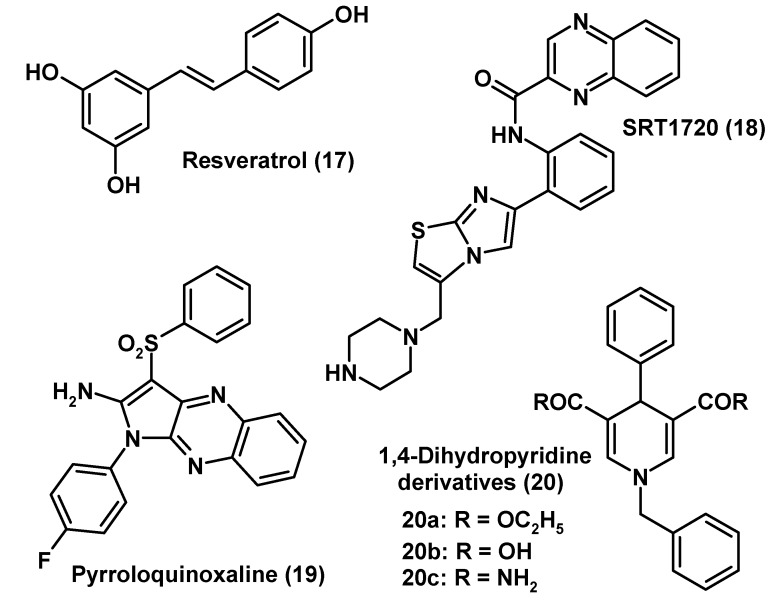
The phytoalexin and SIRT1 ligand, resveratrol (**17**) and several synthetic SIRT activators.

The phytoalexin resveratrol (**17**; Figure 4) has come into the focus in connection with the so-called French paradox [271]. It is present in red wine, but also in peanuts and various medicinal herbs. Without discussing this phenomenon in depth, it can be stated that this phenolic compound is a free-radical scavenger of moderate potency [272,273,274], an antiinflammatory agent [272], and a mitochondrial modulator [147,275,276,277]. Neuroprotection by resveratrol (**17**) has been repeatedly described in neuronal cell cultures, brain slices and animal models, and the effects were associated with various aspects of mitochondrial function, such as NO metabolism, ROS detoxification, activation of AMPK, UCP2, PGC-1α and SIRT1 [143,277,278,279,280,281]. A full consideration of all pertinent findings would exceed the frame of this article. In recent years, resveratrol (**17**) has been mainly discussed as a SIRT1 ligand [143,147,276,281,282,283,284,285]. Although there is an almost general agreement on the activation of SIRT1 by resveratrol, additional actions not excluded, the property of resveratrol (**17**) as a direct SIRT1 ligand has been recently disputed [286]. This would be important in mechanistic terms, but the functional and practical difference may be less relevant. Moreover, resveratrol (**17**) may additionally act via other signaling pathways. For example, the stimulation of AMPK by this compound was shown to proceed via activation of the protein kinase LKB1 (= serine/threonine kinase 11) and concluded to be independent of SIRT1, in this case [143]. Interestingly, LKB1, otherwise known as a tumor suppressor, is also activated by protein kinase Cζ, which responds to peroxynitrite [287], so that the two pathways, the stimulatory one of resveratrol and compensatory one induced by the reactive nitrogen species converge at this point. Activation of the LKB1/AMPK cascade by resveratrol (**17**) has been documented in several non-neuronal tissues or cells, and was reported to be decisive for mitochondrial protection against oxidative stress in HepG2 hepatoma cells [288]. SIRT1-independent actions of resveratrol (**17**) may be also deduced from other findings. Although beneficial effects of resveratrol (**17**) were repeatedly described in Alzheimer’s disease, other neurodegenerative disorders and respective animal models, it was impossible to link genetic SIRT1 variability to these diseases [289]. In conclusion, reducing resveratrol’s actions to SIRT1 activation would be short-sighted, and deny the multiplicity of enzymes and regulatory factors directly or indirectly targeted by this compound [290]. The combination of properties as an antioxidant, an antiinflammatory agent, a modulator of NO and mitochondrial electron flux, and a stimulator of mitochondrial biogenesis should multiply contribute to radical avoidance.

The aging-suppressor function of sirtuins, their neuroprotective potential as well as the connection to mitochondrial functions is beyond doubt, independently of SIRT1 activation by resveratrol (**17**). The triad of reducing free radical formation via modulation of electron flux, of enhancement of energy efficiency and the stimulation of mitochondrial biogenesis seems to be decisive for the health-promoting and antiaging effects. Moreover, these findings are in accordance with a role of SIRT1 as a metabolic sensor and mediator of metabolic adaptation. However, the question remains as to whether the pharmacological efficacy of resveratrol (**17**) is sufficient for mediating these desired effects via SIRT1. This has been tacitly assumed in numerous publications, but may not be generally justified. There are certainly some practical limitations for the use of resveratrol (**17**) for medicinal purposes, concerning solubility, properties of oxidized metabolites and side effects at elevated, pharmacological concentrations. In this regard, its clinical suitability remains to be demonstrated. The same reservation may be appropriate for several other secondary plant metabolites which have been reported to activate SIRT1, too. These are especially butein, piceatannol, fisetin, and quercetin [291].

An alternative was sought in developing potent synthetic SIRT1 activators [292,293]. Some of them are considerably more potent than resveratrol (**17**) [292] and, therefore, could be used at much lower concentrations, which might reduce side effects. A selection of SIRT1 activators, presented in Figure 4, comprises SRT1720 (**18**), pyrroloquinoxaline (**19**), and 1,4-dihydropyridine derivatives **20a-c**. To date, little is known about the pharmacological profile of these compounds, especially with regards to metabolism, deposition, toxicity and other side effects. For all these polycyclic compounds, some caution is especially due as long as their effects on hepatic detoxification mechanisms and possible accumulation in adipose tissue have not been determined. At least, they may be preclinically useful as investigational drugs. The newly developed 1,4-dihydropyridine derivatives **20a-c** act additionally as activators of SIRT2 and SIRT3 [293]. However, it may be questioned whether this non-selectivity is really an advantage. Simultaneous targeting of several sirtuins raises the problem of their functional differences. SIRT2 and SIRT3, as well as SIRT6, were recently reported to be proapoptotic in cerebellar granule neurons [294]. Neuronal cell death was also associated with mitochondrial translocation of SIRT5 [294]. Proapoptotic effects of SIRT3 were also reported for other cells [295]. However, the opposite was reported for cardiomyocytes, in which protection from oxidative stress and cell death was described, and in which SIRT3 was shown to deacetylate Ku70, thereby promoting its interaction with Bax [296]. This can prevent apoptosis [296,297], but Ku70 was also shown to promote the deubiquitinylation of Bax and thereby favor apoptosis [297]. It remains to be clarified to what extent the pro- or antiapoptotic actions of SIRT3 are cell specific or conditional. At moderately elevated levels of SIRT3, its functions as a modulator of mitochondrial metabolism may prevail, as it was shown to deacetylate and activate glutamate dehydrogenase and isocitrate dehydrogenase 2, effects which are not only associated with the regulation of metabolic flux, but also discussed in terms of mitochondrial antioxidant availability [135]. These presumably beneficial effects are in accordance with data on SIRT3 polymorphism showing a relationship to human longevity, especially with regard to the presence of a VNTR enhancer variant that is absent in the oldest individuals [298,299]. The dynamics of SIRT3 expression and activity may, thus, be of great importance, but, as frequently observed, too much of a good may not be good, so that overstimulation of SIRT3 might result in apoptosis. In the future, these considerations, which may also extend to other sirtuins, should not be left out of sight, with regard to both experimental design and development of sirtuin activators.

## Melatonin, Its Analogs and Metabolites

**Figure 5:**
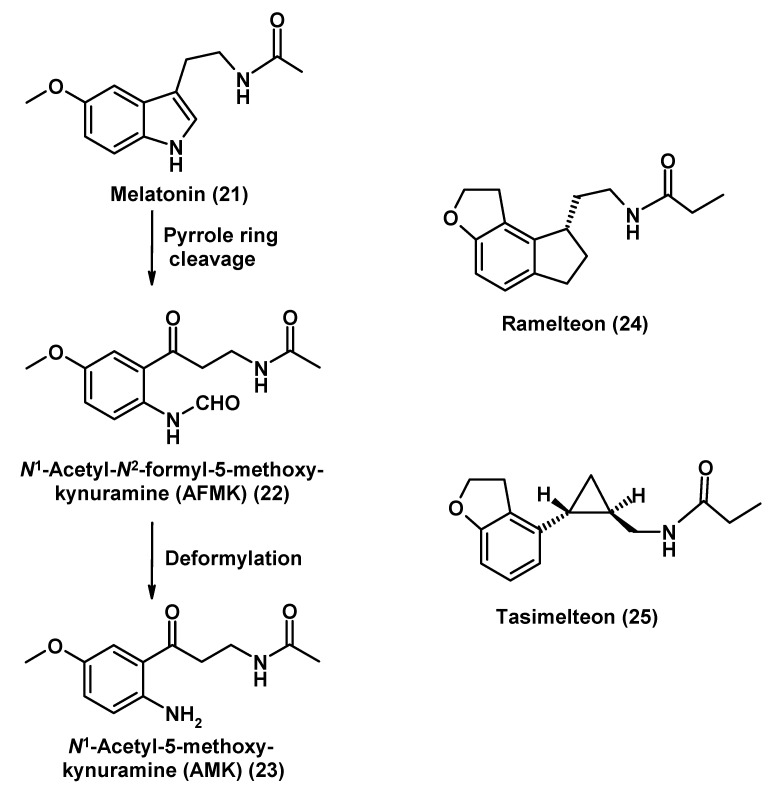
The neuroprotective indoleamine melatonin (**21**), its metabolites formed in the kynuramine pathway, and two synthetic agonists acting via melatonin receptors MT_1_ and MT_2_.

The indoleamine melatonin (**21**; Figure 5) has been discovered as a hormone of the pineal gland. In this role, it acts as chronobiotic, which can reset the circadian master clock and convey information on phase and duration of darkness to numerous organs. In mammals, these actions are widely mediated by the G protein-coupled membrane receptors, MT_1_ and MT_2_. Signaling mechanisms, originally thought to mainly consist of decreases in cAMP, have meanwhile turned out to be much more complex and to comprise, partially in a cell type-dependent fashion, multiple G protein isoforms, activation of phospholipase C and protein kinase C, actions via the MAP kinase and PI3 kinase/Akt pathways and modulation of large conductance Ca^2+^-activated K^+^ and voltage-gated Ca^2+^ channels [300]. This view is, however, still insufficient for understanding the complete physiological role of melatonin (**21**). Apart from the membrane receptors, several other binding sites exist [300,301]. Some of them are nuclear receptors, others are involved in calcium signaling and, more recently, a high-affinity mitochondrial binding site was identified [85,86,261,300,301]. Not all of these proteins are already well characterized with regard to their physiological role.

Contrary to earlier considerations, melatonin does not only act as a hormone [302], but is also formed in numerous extrapineal sites [301,303]. The total amounts of tissue melatonin have been estimated to be several hundred times higher than those found in the pineal gland and in the circulation [303,304]. In the mouse brain, melatonin (**21**) concentrations of up to 0.7 µM were reported [305], data which urgently need confirmation because of their possible implications. From the pineal gland, melatonin (**21**) is not only released to the circulation, but also, at even higher concentrations, via the pineal recess into the third ventricle [306]. Moreover, externally administered melatonin (**21**) was shown to accumulate in mitochondria [307,308], a finding which may be of considerable importance for our understanding of protective mechanisms based on modulation of mitochondrial function.

After the discovery of hydroxyl radical scavenging by melatonin (**21**) [309], the role of this indoleamine was first sought in the direct detoxification of these oxidants. Later, scavenging of various other free radicals has been described, findings which have been repeatedly reviewed [14,304,310,311,312,313,314]. A scavenger cascade involving several reaction products of melatonin (**21**), e.g., its oxidative metabolite *N*^1^-acetyl-*N*^2^-formyl-5-methoxykyuramine (AFMK; **22**) [315], and an additional contribution by the metabolite *N*^1^-acetyl-5-methoxykyuramine (AMK; **23**; Figure 5) [316] allows the detoxification of more than ten free radicals when starting with one melatonin molecule. Nevertheless, considerations based on quantitative relationships between free radicals formed and concentrations of melatonin and other, more abundant antioxidants made readily clear that direct radical scavenging by melatonin (**21**) could be decisive only under some experimental conditions. Next, melatonin (**21**) was found to upregulate several antioxidant enzymes, in particular, glutathione peroxidase, but also enzymes supporting the availability of reduced glutathione, such as glutathione reductase, glucose-6-phosphate dehydrogenase and γ-glutamylcysteine synthase, and to downregulate prooxidant enzymes, such as NO synthases and 5- and 12-lipoxygenases [14,303,304,313,314,317]. These effects were observed in numerous studies, whereas the effects on other antioxidant enzymes were either variable, tissue-specific or limited in their extent. The conviction that these additional effects would not yet be sufficient for explaining the full spectrum of protective effects observed prompted us to suggest a major role of melatonin (**21**) in the attenuation of free radical formation [38]. This should exceed the detoxification of H_2_O_2_, which largely derives from superoxide. In terms of radical avoidance, the following levels of action should be of relevance: (i) adjustment of circadian rhythms in improving internal coordination of oscillations; (ii) antiexcitotoxic effects; (iii) prevention of excessive NO formation leading to elevated peroxynitrite and ETC blockade, and (iv) modulatory effects on mitochondrial electron and proton flux [14,38,317]. Since melatonin (**21**) is a major non-photic synchronizer of circadian rhythms [301], oxidative damage as observed upon perturbations of the internal oscillators [36,37,38] should be attenuated by the indoleamine [14,38].

Antiexcitotoxic effects of melatonin (**21**) have been repeatedly reported, also in conjunction with anticonvulsive actions [317,318,319,320,321,322,323,324]. These sedating effects, which go beyond sleep induction since they are also observed in nocturnal animals, should prevent calcium overload and, thus, calcium-dependent rises in radical formation, thereby contributing to melatonin’s spectrum of protective actions [6]. The anticonvulsive property is obviously mediated by the membrane receptors MT_1_ and/or MT_2_, since they have been also demonstrated with the synthetic, MT_1_/MT_2_-specific agonist ramelteon (**24**; Figure 5) [323]. According to its structure, this non-indolic compound should poorly interact with free-radicals. The antiexcitatory actions of melatonin (**21**) seem to comprise effects on NO formation, especially via inhibition of nNOS [325,326,327,328,329]. Much more work has been published on the downregulation of iNOS, mostly studied in conjunction with sepsis models and in non-neuronal tissues, and more recently with focus on a mitochondrial subform of iNOS (summarized in refs. [86,330,331]). With regard to the central nervous system, these findings may be particularly important in brain inflammatory diseases. Moreover, these studies shed light on preventive actions of melatonin (**21**) at the mitochondrial level. Under the conditions of the animal models used, the suppression of NO formation was associated with elevated activities of ETC complexes, improved ATP formation, maintenance of the mitochondrial membrane potential and reduced formation of superoxide and hydrogen peroxide [308]. The protective actions of melatonin (**21**) regarding the attenuation of NO effects extend to one of its metabolites, AMK (**23**). This substituted kynuramine is a potent scavenger of all NO congeners, thereby forming a stable product that does not re-donate NO [119,332,333,334], and acts additionally as a potent inhibitor of nNOS, which was reportedly effective down to 10^-11^ M [335]. More recently, AMK (**23**) was shown to counteract the induction of the mitochondrial iNOS subform in an MTPT-based model of Parkinson’s disease, thereby reducing oxidative and nitrosative stress [336]. Normalizations of mitochondrial complex I activity observed in the same study with both melatonin (**21**) and AMK (**23**) indicate ETC protection and circumvention of NO/peroxynitrite-induced bottlenecks. In conclusion, melatonin (**21**) as well as AMK (**23**) seem to be capable of attenuating radical formation and preventing damage, as far as this is caused by highly elevated, deleterious concentrations of NO and peroxynitrite. However, an important question remains to be solved, namely, what the influence of either compound will be on moderate, rather favorable levels of NO.

Modulation of mitochondrial electron flux by melatonin (**21**) has been repeatedly described. In many reports, this was, however, related to inflammation and, thus, to NO and peroxynitrite. Many other studies based on administration of rotenone, doxorubicin and MPTP, on models of ischemia/reperfusion or other brain injury are summarized elsewhere [85,86,337,338] and will not be considered here. Moreover, increases in the activities of complexes I, III or IV were usually measured in submitochondrial particles, which might not precisely reflect the *in vivo* situation [85,86]. However, additional information on improvements of ETC function and energy efficiency has come from gerontological studies. Unfortunately, only few of them have been conducted in brain tissue [141,339,340,341]. Much more detailed information is available on other tissues, such as liver, heart and diaphragm, which has been summarized elsewhere [38,85,86,317,337]. These investigations include effects of melatonin in the senescence-accelerated mouse strain SAMP8, which can be compared with the normally aging strain SAMR1 sharing the same genetic background. Collectively, results show the same changes. Especially at advanced age, SAMP8 animals exhibit reductions in the respiratory control index (RCI), in state 3 and in dinitrophenol-uncoupled respiration, in the ADP/oxygen ratio, frequently also in ATP concentrations, and decreases in the activities of complexes I and IV. The latter finding contrasts with earlier results in normally aging mice, which exhibited rises in complex IV activity [339]. The differences may be attributed to variations in the use of submitochondrial particles. Rises in state 4 respiration reported in old SAMP8 mice [137] should be seen in line with rises in complex IV activity, as observed in normal aging mice [339]. In the situation of aging, enhanced complex IV activity does not necessarily indicate an advantage, but may reflect a compensatory reaction caused by impaired energy efficiency. The respiratory effects were usually associated with declines in the GSH/GSSG ratio and glutathione peroxidase activity, with rises in lipid peroxidation and protein carbonyl levels. Regardless of this detail, all reports unanimously describe the reversion of age-related mitochondrial changes by melatonin (**21**). Moreover, the indoleamine was found to increase half-life and maximal lifespan in SAMP8 mice [342].

Although these studies concordantly emphasize the support of mitochondrial function by melatonin (**21**), the interpretations may be either preliminary or incomplete. Of course, the antioxidant and antinitrosant actions of the indoleamine contribute to protection. This should include the avoidance of cardiolipin peroxidation, thereby favoring respirasomal integrity [341]. However, additional mitochondrial effects have to be considered. A direct inhibition of the mitochondrial permeability transition pore by melatonin (**21**) is observed at elevated concentrations [343], and presumably based on a low-affinity binding site, should be of importance in experiments on prevention of apoptosis. Interpretations concerning effects in the lower pharmacological range, as by administration via the drinking water, would require other interpretations. One idea had been that melatonin (**21**) might participate in a kind of redox cycling by interacting with components of the ETC and contributing to an electron shuttle [14,38]. This possibility was also discussed for the melatonin metabolite AMK (**23**) [14,38], which also exerts protective effects in mitochondria [344]. This interpretation would require further substantiation. Another recent finding concerns the existence if a high affinity binding site with a K_d_ of 150 pM, localized according to inhibitor studies at the amphipathic ramp of complex I. These results have not yet been published in detail, but were cited a couple of times [84,85,86,317]. If melatonin binding to this site is not related to electron exchange reactions, as previously assumed [14,38], its presence would imply a regulatory role at the first control point of the ETC and should, therefore, be assumed to modulate electron flux. This possibility would go beyond classic mitochondrial protection by an antioxidant and antinitrosant agent, but could likewise contribute to antioxidative effects by metabolic adaptation.

Another kind of metabolic adaptation, with respective consequences for respiratory capacity, energy efficiency and electron leakage, might be achieved by stimulation of mitochondrial biogenesis. We had previously suggested a relationship between melatonin (**21**) and sirtuins [84], an assumption that has recently gained some support. In SAMP8 mice, a few, rather preliminary data showed upregulation of SIRT1 by melatonin [345]. More detailed studies have now demonstrated that melatonin (**21**) favors the hippocampal expression of SIRT1 in a model using sleep-deprived rats [346]. In another investigation, effects of melatonin (**21**) were compared in neuronal cultures from young and aged rats [347]. Melatonin stimulated SIRT1 expression in the aged neurons to levels approximating those from young rats and caused enhanced deacetylation of various SIRT1 substrates, such as PGC-1α, FoxO1, NFκB, and p53, effects which were largely reverted by the SIRT1 inhibitor sirtinol [347]. Although this has not yet been demonstrated directly, the melatonin-induced deacetylation of PGC-1α strongly suggests that a long-term treatment with the indoleamine would stimulate mitochondrial biogenesis.

Finally, another connection between melatonin (**21**) and SIRT1 seems to exist, which may deserve further attention. SIRT1 was shown to modulate chromatin remodeling via the circadian clock gene protein CLK. Thereby, it seems to directly influence at least peripheral oscillators by interacting with the CLK/BMAL1 complex [215,348]. Remodeling of chromatin represents a necessity of circadian gene expression and is also influenced by melatonin (**21**), the major non-photic synchronizer. Relative melatonin deficiency, as occurring during aging, causes circadian dysregulations [349]. Pronounced rhythms in metabolism exist in numerous organs including the brain, and consequently lead to periodic radical generation [38]. To reduce oxidative stress, a delicate internal coordination of rhythms is required [36,37,38]. Hence, the convergence and eventual interdependence of melatonin (**21**) and sirtuin pathways might be of high interest in terms of aging and radical avoidance. The effects on chromatin structure should lead to numerous secondary changes in circadian functions, including mitochondrial metabolism.

## Conclusions

The attenuation of free radical formation should be regarded as an important contribution to the protection from damage by reactive oxygen and nitrogen species. The more efficient the mechanisms of radical avoidance are working, the less detoxification of radicals is required, so that higher quantities of natural radical scavengers are preserved and available when needed.

The natural and synthetic compounds discussed differ with regard to the mechanisms of radical avoidance, however, with a considerable overlap and various cross-links. Global regulation mechanisms of radical avoidance related to the internal coordination of circadian rhythms are mainly associated with the chronobiotic actions of melatonin (**21**). This should be also assumed for synthetic melatoninergic agonists, such as ramelteon (**24**) or tasimelteon (**25**). Antiexcitatory activities, which reduce radical formation by counteracting glutamatergic signaling, preventing calcium overload and excessive NO production, are characteristic properties of kynurenic acid (**12**), which can be made available to the central nervous systems by administration of indole-3-pyruvic acid (**14**), of memantine (**15**), L-theanine (**16**), melatonin (**21**) and ramelteon (**24**). Antiinflammatory actions have been described for many of the compounds discussed, such as PBN (**1**), thiazolidinediones like rosiglitazone (**8**) and pioglitazone (**9**), resveratrol (**17**), melatonin (**21**) and its metabolite AMK (**23**). These effects are frequently associated with decreases in the formation of NO and peroxynitrite, changes which are also crucial to avoiding impairment of ETC functions and electron dissipation caused by malfunctioning respirasomes. Attenuation of NO production is also relevant under other various pathophysiological conditions and can be decisive in preventing neuronal death. Pertinent effects have been described for nitrones (**1–7**), kynurenic acid (**12**), leptin, resveratrol (**17**), melatonin (**21**) and AMK (**23**). Enhanced radical formation, as induced by various mitochondrial toxins, is counteracted by resveratrol (**17**), melatonin (**21**), AMK (**23**), L-theanine (**16**) and presumably also leptin. Physiological modulation of mitochondrial metabolism is possible either via upregulation of UPC2, as shown for leptin and the nutritional factor resveratrol (**17**), or via mechanisms involving SIRT1, AMPK, PGC-1α, and PPARγ, as observed with resveratrol (**17**), melatonin (**21**) and leptin. The last-mentioned signaling pathways can also initiate mitochondrial biogenesis. They are additionally involved in the adaptation to nutrient availability and can be, thus, influenced via calorie restriction. Nutrition and respiration are also subject to circadian variations and, in this regard, again target to melatonin (**21**) and presumably synthetic melatoninergic agonists as well, such as ramelteon (**24**) and tasimelteon (**25**).

The various compounds discussed do not only differ with regard to their cellular action spectra, but also in terms of suitability for practical purposes. As requirements of neuroprotection are largely related to aging and aging-associated disorders, the long-term tolerability is of premier importance. Leptin cannot be directly given as a drug for neuroprotection in the context of aging, and properties of leptin mimetics crossing the BBB remain to be studied in the future. Long-term safety may be critical with nitrones and, perhaps, with thiazolidinediones, presumably less with memantine and indole-3-pyruvic acid. The long-term safety of AMK (**23**) has not been tested, but the observed adduct formation with tyrosyl residues, with possible immunological consequences [350], may not be in favor of prolonged treatments. At reasonable concentrations, the food constituents L-theanine (**16**) and resveratrol (**17**) as well as melatonin (**21**) should be safe. Whether this is also valid for elevated, pharmacological amounts of resveratrol, may not be certain. Very high doses of melatonin (**21**) have been shown to be safe in a study on ALS patients [351]. Synthetic melatonin agonists, such as ramelteon (**24**) and tasimelteon (**25**), are widely devoid of side effects during short-term treatment, but require further clinical studies on extended exposures [352,353].

The search for agents suitable in radical avoidance should be continued and is worth the effort. As pharmaceutical companies tend to neglect natural compounds, in favor of patentable synthetic drugs, a few desirable properties of such substances shall be emphasized. Apart from being non-toxic after prolonged exposure, they should (i) not interfere with the cytochrome P_450_ enzymes to minimize drug interactions; (ii) be superior, in terms of bioavailability, to the natural compounds, especially short-lived indolic and phenolic substances with radical scavenging properties; (iii) not accumulate in adipose tissue because of high lipophilicity, (iv) target mitochondria either directly as amphiphilic modulators or indirectly by signaling, e.g., via SIRT1, PGC-1α *etc*., and (v) not generate metabolites with undesired properties. However, it will not be easy to develop drugs which combine these requirements with the orchestrating, pleiotropic regulatory effects of a natural compound like melatonin.
